# Dual Pathology: A Case of Concurrent Lymphocytic Esophagitis and Crohn’s Disease

**DOI:** 10.7759/cureus.106366

**Published:** 2026-04-03

**Authors:** Maryam Al Khalifa, Madhawi Albuainain, Sanad Sanad, Abdulla Darwish, Mohamed Awadh, Dana Alsharqi

**Affiliations:** 1 Medicine, Bahrain Defence Force Hospital - Royal Medical Services, Riffa, BHR; 2 Internal Medicine, Bahrain Defence Force Hospital - Royal Medical Services, Riffa, BHR; 3 Gastroenterology, Bahrain Defence Force Hospital - Royal Medical Services, Riffa, BHR; 4 Pathology and Laboratory Medicine, Bahrain Defence Force Hospital - Royal Medical Services, Riffa, BHR

**Keywords:** crohn’s disease (cd), dysphagia, esophagitis, inflammatory bowel disease, lymphocytic esophagitis

## Abstract

Crohn’s disease (CD) is a chronic, systemic disease characterized by transmural inflammation of the gastrointestinal (GI) tract. Patients can present with a range of GI and extraintestinal features depending on the areas involved. Esophageal involvement can be a direct result of Crohn’s or other co-existing diseases. Determining the cause of esophagitis in CD is important as it affects management and prognosis. Lymphocytic esophagitis (LE) is an example of a disease that can cause esophagitis on a background of CD. Patients presenting with the two conditions simultaneously are relatively rare. We present a case of LE and CD in a young male patient.

## Introduction

Crohn’s disease (CD) is a chronic inflammatory disease that can affect any part of the digestive tract. The terminal ileum is the most commonly affected site, while the upper gastrointestinal (GI) tract including the esophagus is less commonly involved [1]. CD can directly affect the esophagus or can be associated with other inflammatory conditions of the esophagus including eosinophilic esophagitis and upper GI CD [1]. Esophageal involvement in CD typically presents with ulcers that result in dysphagia and odynophagia. On histopathology, transmural lymphocytic infiltration can be seen, with or without granulomas [2]. Another type of esophagitis that is associated with, but not caused by, CD is lymphocytic esophagitis (LE) [3]. Determining the cause of the esophagitis and differentiating it from other inflammatory causes of the esophagus are important in order to administer appropriate treatment. Currently, there is limited evidence regarding the treatment options for LE besides symptomatic relief. 

## Case presentation

A 25-year-old man with no known medical history was referred to the gastroenterology service with colicky abdominal pain, anal pain, bloody diarrhea, and oral ulcers for the past three months. He also noticed a 12-kilogram involuntary weight loss over two months, as well as generalized joint pains and lower back pain. Physical examination was remarkable for mild right lower quadrant abdominal tenderness and evidence of a perianal fistula on rectal examination, but was otherwise unremarkable. 

**Figure 1:**
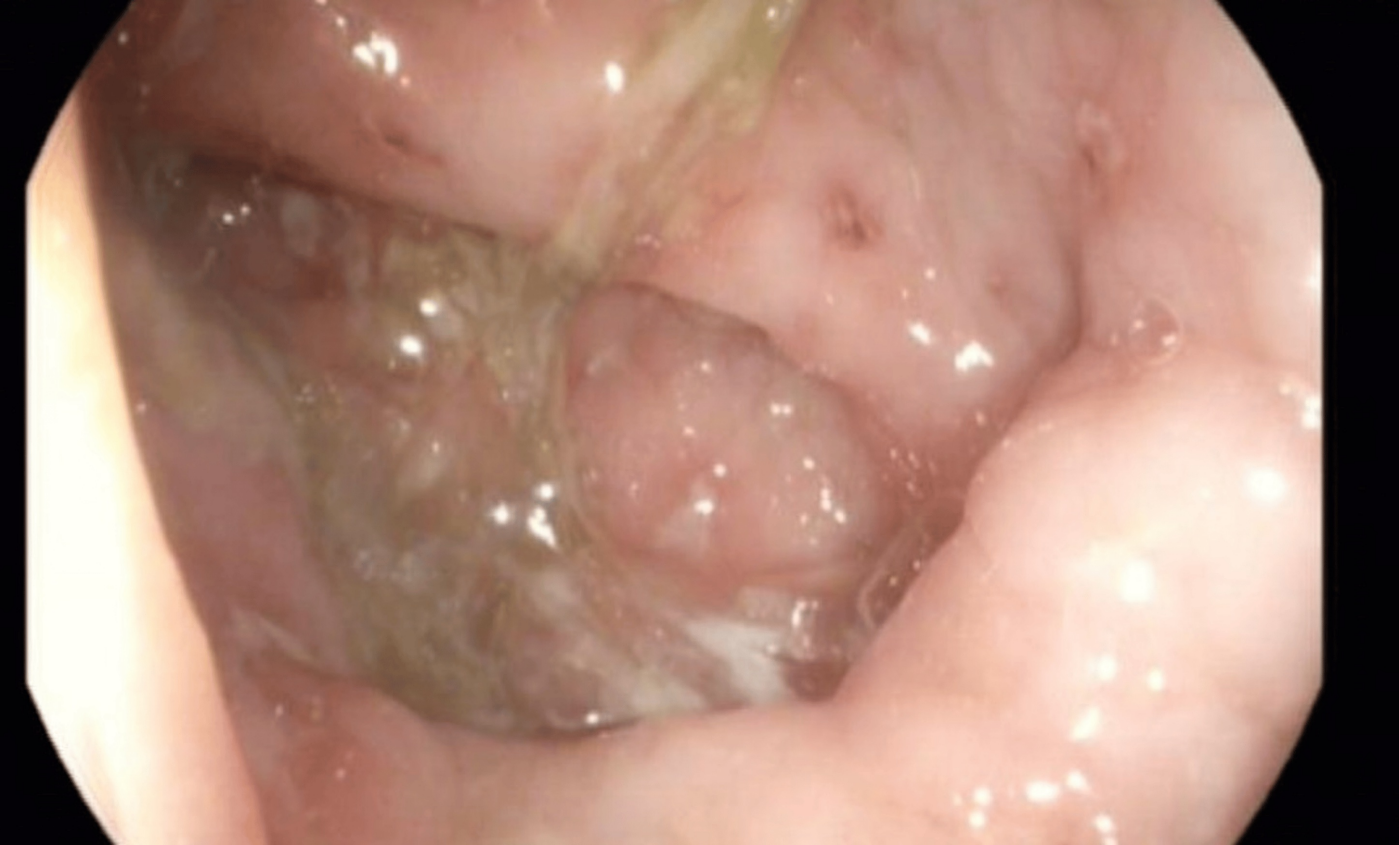
Colonoscopy showing mild erythema and cobblestoning of the colonic mucosa.

**Figure 2:**
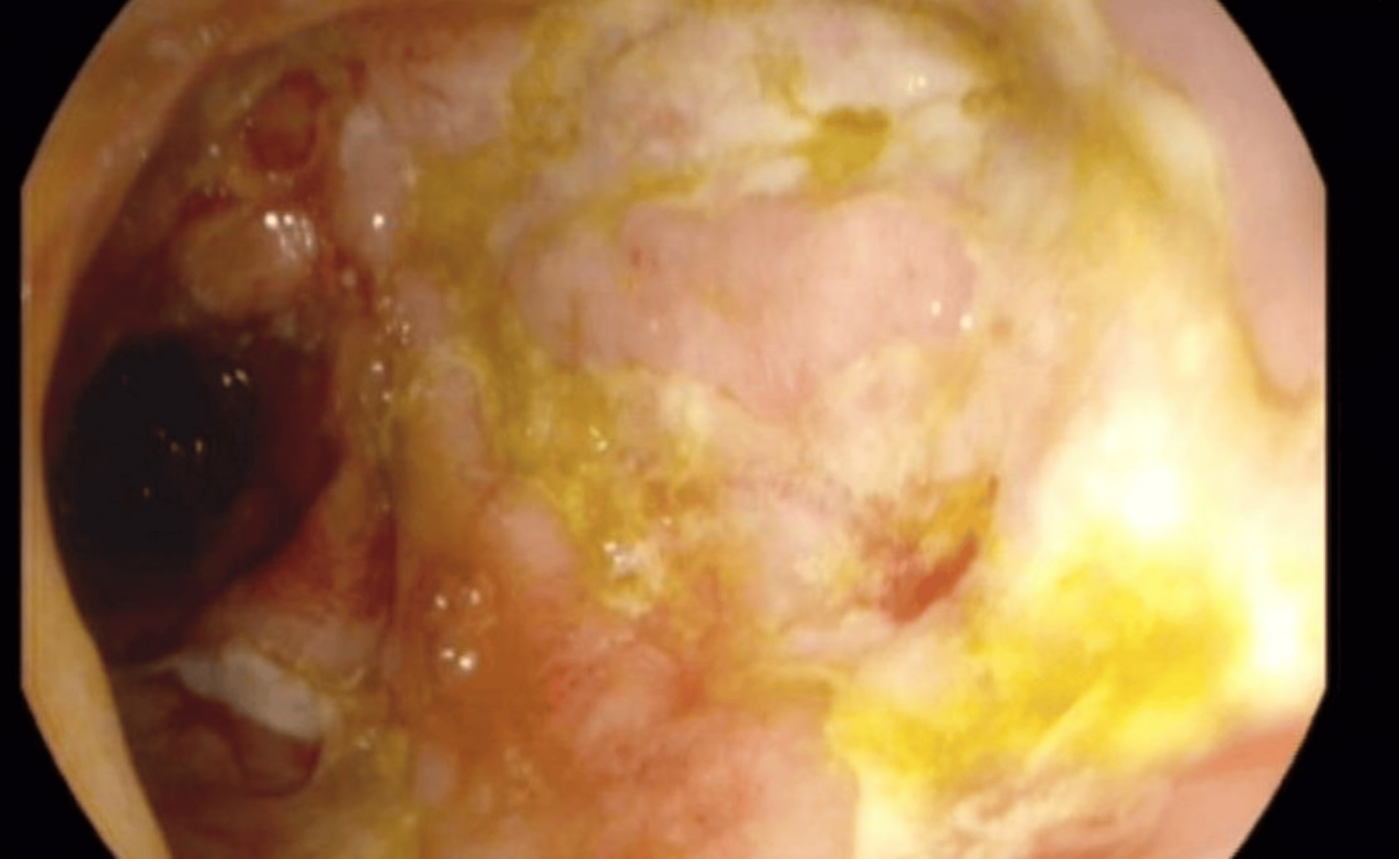
Colonoscopy showing severe cobblestoning and ulceration of the colonic mucosa.

An esophago-gastro-duodenoscopy (EGD) and colonoscopy were performed. EGD showed a normal esophagus, gastritis and duodenitis, and colonoscopy showed a perianal fistula, erythema, cobblestoning, and ulceration progressing in severity towards the right colon and terminal ileum. The disease was most severe in the terminal ileum (Figures 1, 2). Colonic and terminal ileal biopsies were consistent with active CD, while gastric biopsies showed gastritis. Lab results are included in Table 1. 

**Table 1:** Results from the patient's blood work including complete blood count, urea and electrolytes, C-reactive protein, and liver function tests.

Test	Patient’s Results	Reference Range
White blood cells	7.93 x10^9^/l	4-11 x10^9^/l
Hemoglobin	9.9 g/dl	13-18 g/dl
Mean corpuscular volume (MCV)	78.9 fl	80-100 fl
Mean corpuscular hemoglobin (MCH)	25.3 pg	27-32 pg
Platelets	555 x10^9^/l	150-450 x10^9^/l
Sodium	135 mmol/l	136-145 mmol/l
Potassium	4.07 mmol/l	3.5-5.1 mmol/l
Chloride	99.2 mmol/l	98-107 mmol/l
Carbon dioxide	24.4 mmol/l	22-29 mmol/l
Creatinine	70.1 umol/l	62-106 umol/l
Urea	3.63 mmol/l	2.76-8.07 mmol/l
Total bilirubin	20.6 umol/l	0-21 umol/l
Direct bilirubin	7.71 umol/l	0-5 umol/l
Alkaline phosphatase (ALP)	96 u/l	40-129 u/l
G-glutamyltransferase (GGT)	13.7 u/l	0-60 u/l
Alanine aminotransferase (ALT)	41.6 u/l	0-50 u/l
Aspartate aminotransferase (AST)	51.4 u/l	0-50 u/l
C-reactive protein (CRP)	52.2 mg/l	0-5 mg/l

While awaiting biologic therapy, the patient was commenced on prednisolone 40 milligrams (mg) tapered down by 5mg every week and azathioprine 50mg, with a target dose of 150mg a day. He developed a reaction to the azathioprine in the form of a severe headache, rash, and deranged liver function tests, necessitating cessation of the drug. Infliximab was subsequently started at 10 mg/kg with an excellent clinical, biochemical, and endoscopic response both in terms of GI and extra-intestinal disease. The patient also received one gram of intravenous iron to address his anemia (Table 1).

**Figure 3:**
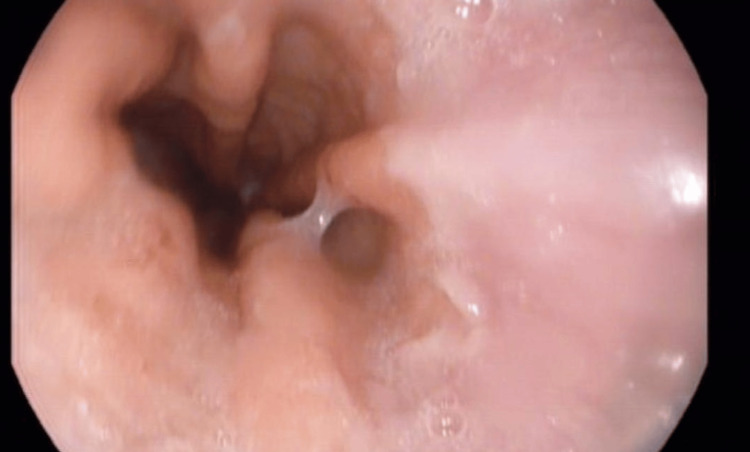
EGD showing a normal-appearing gastro-esophageal junction. EGD: Esophago-gastro-duodenoscopy

**Figure 4:**
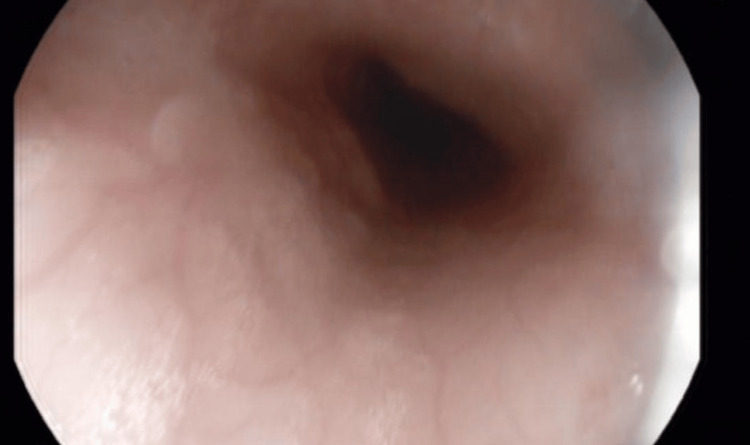
EGD showing normal esophageal mucosa. EGD: Esophago-gastro-duodenoscopy

**Figure 5:**
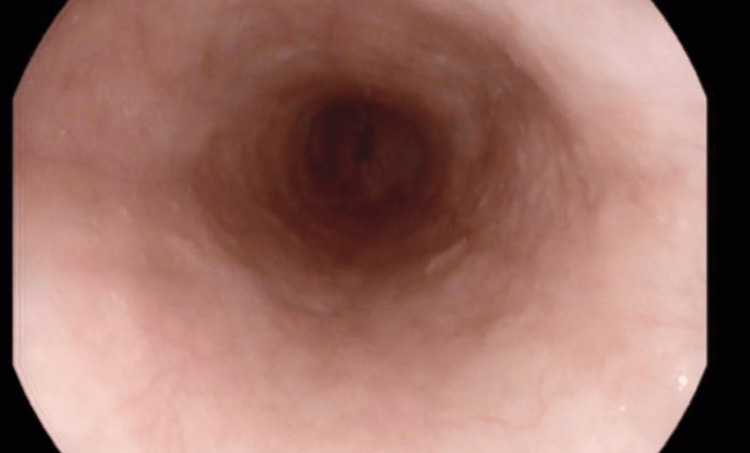
EGD showing normal esophageal mucosa. EGD: Esophago-gastro-duodenoscopy

Two months into commencing anti-TNF therapy, the patient started experiencing progressively worsening dysphagia to solids. He had no odynophagia, reflux symptoms, or vomiting, and he was continuing to gain weight with complete remission with regard to his previous symptoms. During this time, he was not taking any new dietary supplements or medications. On physical examination, there was no goiter, and he had no evidence of oral candidiasis. An EGD was done showing normal-appearing esophageal mucosa, with no rings, strictures, or other features suggestive of eosinophilic esophagitis. There was no evidence of esophageal candidiasis and no esophageal ulcers or other abnormalities noted, ruling out upper GI CD (Figures 3-5).

**Figure 6:**
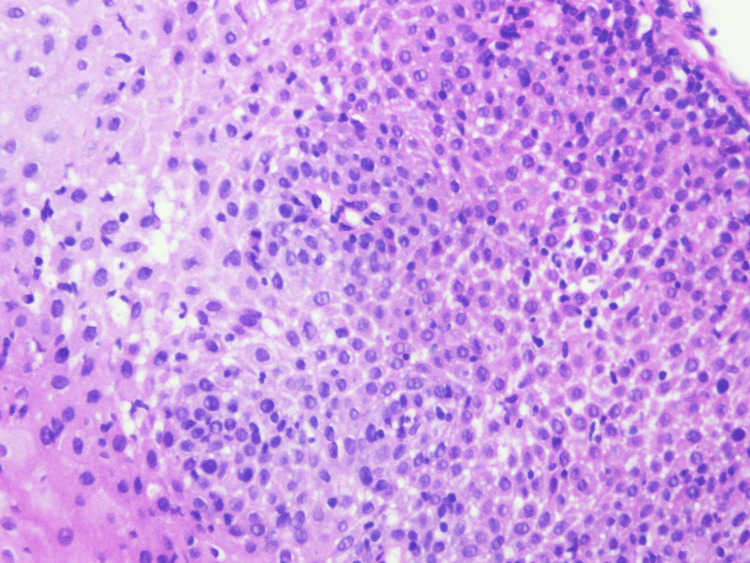
Histopathology image from esophageal biopsy showing lymphocytic infiltrate (magnified 40x).

**Figure 7:**
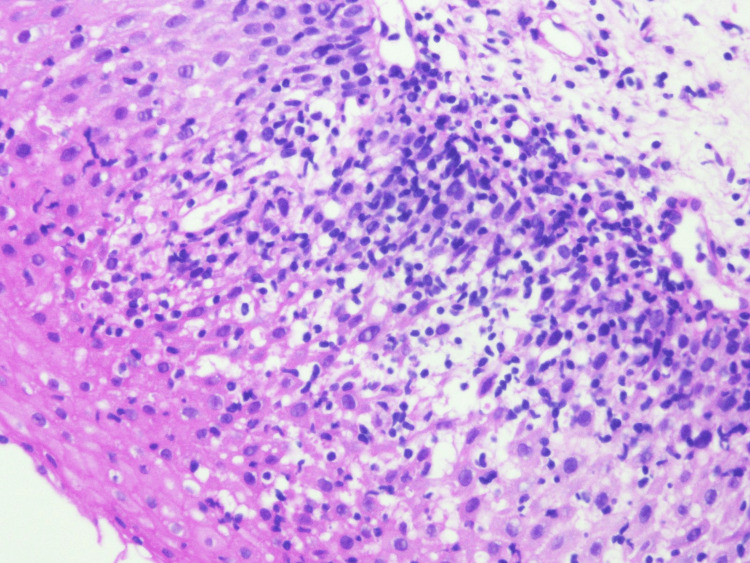
Histopathology image from esophageal biopsy showing lymphocytic infiltrate (magnified 40x).

**Figure 8:**
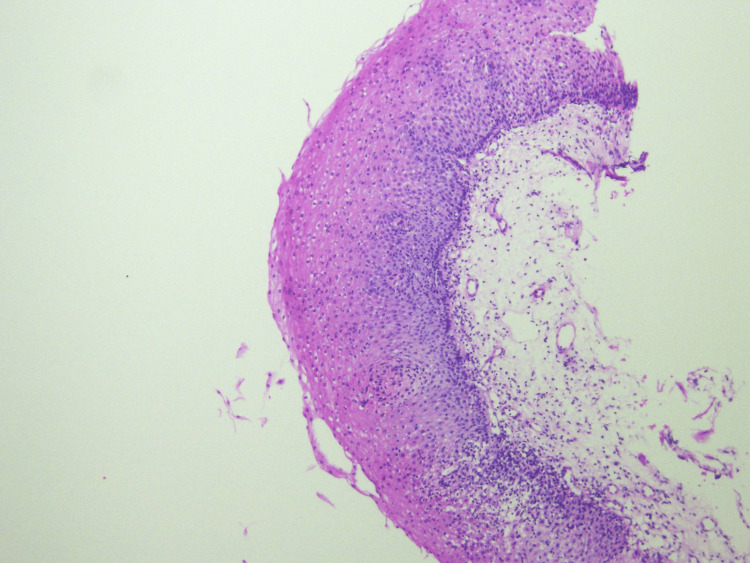
Histopathology image from esophageal biopsy showing lymphocytic infiltrate (magnified 20x).

Biopsies were taken from the middle and lower thirds of the esophagus showing basal cell hyperplasia with spongiosis and marked intraepithelial lymphocytes (>60 per high-power field) in keeping with LE (Figures 6-8). There was no increase in eosinophils, infectious pathogens, dysplasia, or malignancy noted on histopathology. Based on the presentation of dysphagia with normal endoscopy findings combined with the histopathology results, a diagnosis of LE was made. Pantoprazole 40 milligrams per day was then started with complete resolution of symptoms within roughly two months. The patient currently remains asymptomatic and continues to receive infliximab and pantoprazole.

## Discussion

We present a case of concomitant CD and LE in a young male. CD is a chronic, inflammatory disorder of the GI tract characterized by transmural, segmental inflammation. All parts of the GI tract from the mouth to the anus can be affected [1]. The etiology of CD is thought to be multifactorial involving interaction between environmental and genetic factors [4,5]. Upper GI tract involvement is less commonly seen in adults, but more so in the pediatric population [6]. The prevalence of esophageal involvement in adults is reported to be between 3.3% and 6.8% compared to a range of 7.6% and 17.6% in children [5]. However, this value is likely an underestimation as upper endoscopy is performed less commonly in adult patients relative to pediatric patients [5]. Common endoscopic findings in esophageal CD include strictures, fistulas, bamboo joint-like appearance, and aphthous or longitudinal ulcers [5]. 

Dysphagia in the CD patient has multiple differentials. Upper GI CD should be ruled out [5]. Infections due to immunosuppressive therapy including esophageal candidiasis, and concomitant immune disorders such as eosinophilic esophagitis, among others, are also within the differential [4,5]. Ultimately, the diagnosis is based on upper GI endoscopy and histopathologic evaluation. 

LE is a more recently recognized chronic disease characterized by lymphocytic infiltration on esophageal biopsies (>20 intra-epithelial lymphocytes per high-power field). It is more common in women and commonly presents in the fifth to sixth decades of life, with the median age being 51 years. It is also more prevalent in Caucasians [6]. Most cases of LE present with dysphagia. Other less common presentations include odynophagia, heartburn, nausea, as well as chest and abdominal pain [6,7]. Apart from inflammatory bowel disease, it seems to be associated with other conditions including gastro-esophageal reflux disease and hypothyroidism [6]. Endoscopic findings can be variable and include rings, strictures, and esophagitis, with lesions most commonly being found in the mid-esophagus [7]. A third of patients have normal mucosa on endoscopy [7]. Biopsy confirmation is essential to rule out eosinophilic esophagitis and upper GI CD. 

Currently, there is no consensus as to the management of LE. Symptomatic relief can be achieved with proton pump inhibitors (PPIs) or topical steroids such as swallowed fluticasone [3]. Endoscopic dilatation can also be used alongside medical therapy for stricturing disease [3]. However, there is an overall lack of evidence regarding the efficacy of these treatments [8].

While previous studies have shown an association of LE and CD in pediatric patients, the evidence on the association between the two in adults is less robust [7,9]. A case report published in 2024 and a systematic review published in 2019 [10,11] present cases in adults, but both of these were middle-aged women rather than a young male, as in our case. Both showed more severe findings including stricturing, and required endoscopic dilatation, which our patient did not require. Another 2024 paper by Lavette et al. [12] reviewed cases of LE and concluded that there was, in fact, an association with CD, which was stronger in children but also existed in adults. It is currently unclear as to what causes this association, but risk factors such as medications and genetic predisposition for autoimmune disease may play a role [12].

Looking back at our case, our patient was not in the typical age range of other cases published in the literature and had milder symptoms and a normal endoscopic appearance, with an excellent response to PPI therapy. This case report adds to the sparse literature on LE in the setting of CD. The awareness of the approach to dysphagia in CD, and of LE in particular, is important, as this condition remains relatively under-reported. 

## Conclusions

LE is an underdiagnosed disease that should be suspected in patients with dysphagia with no clear cause. It should especially be considered in patients with CD with symptoms of upper GI involvement. A higher index of suspicion is needed in pediatric patients, as there is a greater association than in adults. Biopsies should be taken to differentiate LE from other diseases that present in a similar way. 
